# Curcumin Extraction, Isolation, Quantification and Its Application in Functional Foods: A Review With a Focus on Immune Enhancement Activities and COVID-19

**DOI:** 10.3389/fnut.2021.747956

**Published:** 2021-09-21

**Authors:** Soubhagya Tripathy, Deepak Kumar Verma, Mamta Thakur, Ami R. Patel, Prem Prakash Srivastav, Smita Singh, Alok Kumar Gupta, Mónica L. Chávez-González, Cristobal Noe Aguilar, Nishant Chakravorty, Henu Kumar Verma, Gemilang Lara Utama

**Affiliations:** ^1^Agricultural and Food Engineering Department, Indian Institute of Technology Kharagpur, Kharagpur, India; ^2^Department of Food Technology, School of Sciences, ITM University, Gwalior, Madhya Pradesh, India; ^3^Division of Dairy Microbiology, Mansinhbhai Institute of Dairy & Food Technology-MIDFT, Gujarat, India; ^4^Department of Life Sciences (Food Technology), Graphic Era (Deemed to Be) University, Dehradun, India; ^5^Department of Nutrition and Dietetics, University Institute of Applied Health Sciences, Chandigarh University, Chandigarh, India; ^6^Division of Post-Harvest Management, ICAR-Central Institute for Subtropical Horticulture (Ministry of Agriculture and Farmers Welfare, Government of India), Lucknow, India; ^7^Bioprocesses Research Group, Food Research Department, School of Chemistry, Universidad Autonoma de Coahuila, Saltillo, Mexico; ^8^School of Medical Science and Technology, Indian Institute of Technology Kharagpur, West Bengal, India; ^9^Department of Immunopathology, Comprehensive Pneumology Center, Institute of Lungs Biology and Disease, Munich, Germany; ^10^Faculty of Agro-Industrial Technology, Universitas Padjadjaran, Sumedang, Indonesia; ^11^Center for Environment and Sustainability Science, Universitas Padjadjaran, Bandung, Indonesia

**Keywords:** curcumin, separation methods, human immune system, immunological activity, human health, functional food, COVID-19

## Abstract

An entirely unknown species of coronavirus (COVID-19) outbreak occurred in December 2019. COVID-19 has already affected more than 180 million people causing ~3.91 million deaths globally till the end of June 2021. During this emergency, the food nutraceuticals can be a potential therapeutic candidate. Curcumin is the natural and safe bioactive compound of the turmeric (*Curcuma longa* L.) plant and is known to possess potent anti-microbial and immuno-modulatory properties. This review paper covers the various extraction and quantification techniques of curcumin and its usage to produce functional food. The potential of curcumin in boosting the immune system has also been explored. The review will help develop insight and new knowledge about curcumin's role as an immune-booster and therapeutic agent against COVID-19. The manuscript will also encourage and assist the scientists and researchers who have an association with drug development, pharmacology, functional foods, and nutraceuticals to develop curcumin-based formulations.

## Introduction

**Figure 1:**
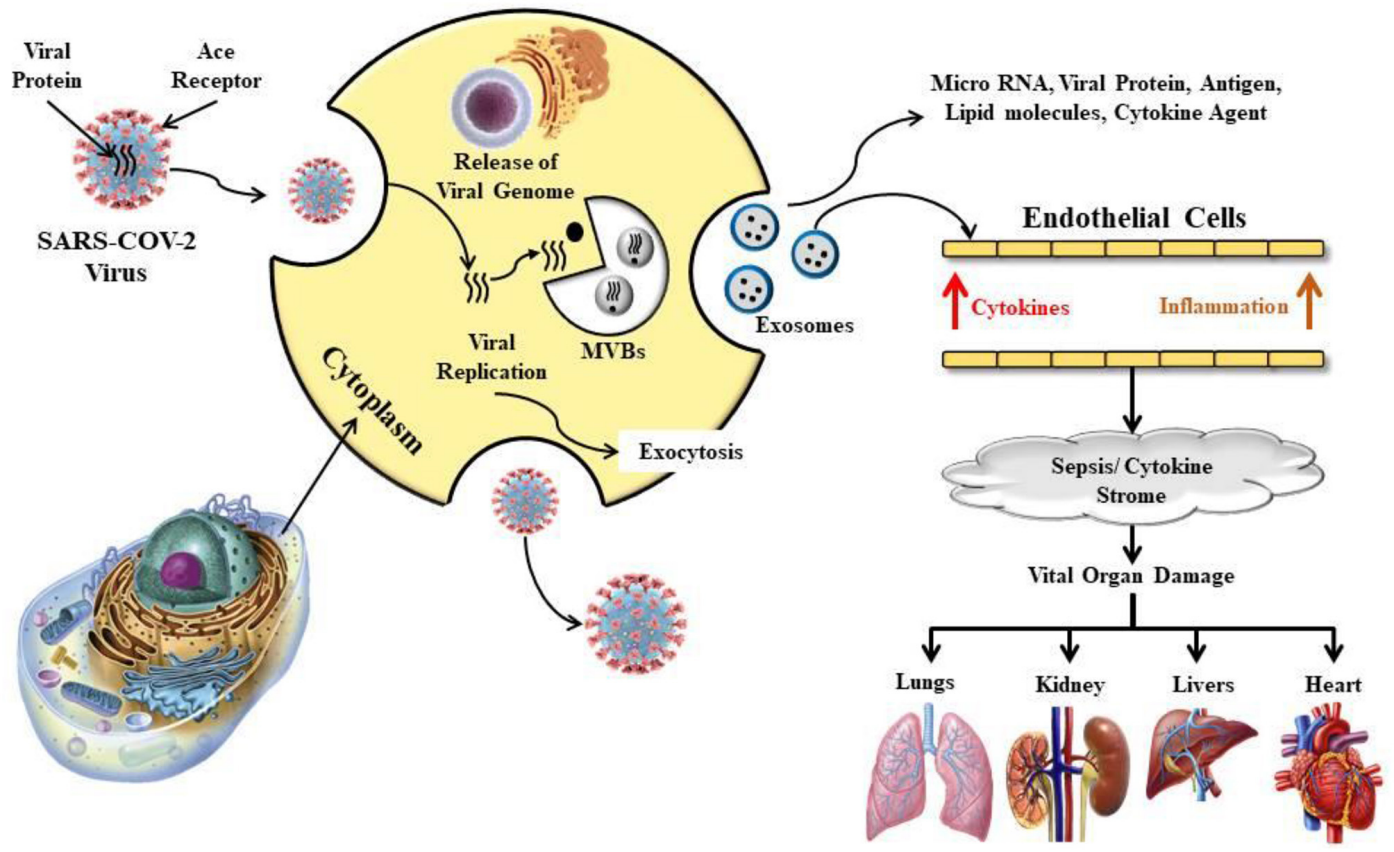
Schematic presentation on the molecular mechanisms involved in the SARS-COV-2 virus infection in the human respiratory system, as well as the detrimental effects on other important organs of the human body.

The human immune system is the key to our body's defense against invading pathological microorganisms, extrinsic agents and conditions like cancer. An individual with a weakened immune system is more likely to suffer from infections caused by pathogens like bacteria, viruses, parasites and fungi (1). An outbreak of new viral infection was first recorded in China at the end of December 2019, which has wreaked havoc across the globe since then. This latest virus, known as Severe Acute Respiratory Syndrome Coronavirus 2 or SARS-CoV-2, is a highly infectious pathogen with high levels of morbidity and mortality, leading to a global pandemic (2–5). Coronaviruses are huge, pleomorphic but mostly spherically enveloped, non-segmented, positive-stranded RNA viruses i.e., +ssRNA having 5′ -cap structure and 3′-poly -A tail, and possess the largest genome (27–32 kb) in all RNA viruses (6). The infectious SARS-CoV-2 varies in size from 50 to 200 nm (dia) and is comprised of the following major structural proteins: (i) envelope (E), (ii) membrane (M), (iii) nucleocapsid (N), and (iv) spike (S) (trimeric) (6, 7). They contain protrusions (80–120 nm dia) of glycoproteins above the surface (8). M protein and E protein are involved in the virus assembly whereas the S protein creates the big projections above the surface (9, 10). Through these protrusions, S protein attaches to the cell membrane of a host by targeting the angiotensin-converting enzyme 2 (ACE2) receptors of the host cell, mainly found in the respiratory epithelium and alveoli of the lungs (11, 12). ACE2 protein is also expressed in several organs of humans including kidney and intestine, which are therefore, the main targets of CoV (13). The molecular mechanisms of SARS-COV-2 virus infection in the human respiratory system, as well as the harmful consequences on other vital organs of the human body have been presented in Figure 1.

The most likely cause of the SARS-CoV-2 infection-related symptoms (COVID-19) is attributed to its impact on the human immune response. Like SARS and MERS, this coronavirus also affects the host's innate immune system and causes elevated levels of pro-inflammatory cytokines like IL1B, IFN-γ, IP10, MCP1, MIP1A, and TNF-α and also reduces the body's lymphocyte count, thus suppress in the adaptive immune system (14, 15). Although supportive management guidelines and protocols are being regularly updated, no anti-viral drug having specific activity against SARS-CoV-2 has been identified yet. Presently, several drugs across the globe are in clinical trials for the management of COVID-19 and convalescent plasma therapy could be a future alternative for serious patients (15–18). International and national organizations dedicated to public health, advocate certain practices to prevent the spread of this novel virus-like, such as maintaining social distance, regular handwashing with soap or alcohol sanitizer, the use of an approved face mask to cover the mouth and nose, and the strengthening the body's immune system (3, 4, 15, 16, 19). While there are several medicines available in the market which claim to be immunoboosters; however, the most healthy, safe and cost-effective way to strengthen our immune system is by consuming healthy functional foods. Functional food can therefore play an important role in improving human immunity against deadly viruses like SARS-CoV-2.

The term “functional food” can be described as “food with one or more target beneficial effects on the human body other than nutritional effects, and food should reduce the risk of any disease or improve human health,” as Functional Food Science in Europe (FUFOSE) indicated (20). Functional foods are either natural food or one or more additional compounds that can boost consumer efficiency at any age or in a particular age group (21–24). Furthermore, there is scientific evidence of a beneficial impact on human health in the functional food produced by incorporating plant parts that have known or unknown bioactive substances (23, 25). The biological properties of such bioactive compounds can influence human health as antioxidant activity, anti-inflammatory activity, antimicrobial activity, anti-diabetic activity, and anticancer activity, etc. (5, 25, 26). Several bioactive components like flavonoids, phenolic acid, alkaloids, etc. are present in plants such as *Allium sativum, Curcuma longa, Ocimum tenuiflorum, Phyllanthus emblica, Piper nigrum, Tinospora cordifolia*, etc. which contribute to their therapeutic characteristics, as presented in Table 1 (25, 51, 60–63). As found in previous studies, these plants are immunologically active, such as enhanced antibody production and macrophage mobility, cell-mediated immunity (64). Therefore, these plants can be included in food formulations to develop functional foods, due to their bioactive substances and medicinal activities.

**Table 1:** Summary of bioactive compounds present in different food samples along with their mode of action.

**Food sample**	**Particular bioactive compound**	**Immunity against**	**Bioassay**	**Doses**	**Mode of action**	**References**
Amla (*Phyllanthus emblica*) 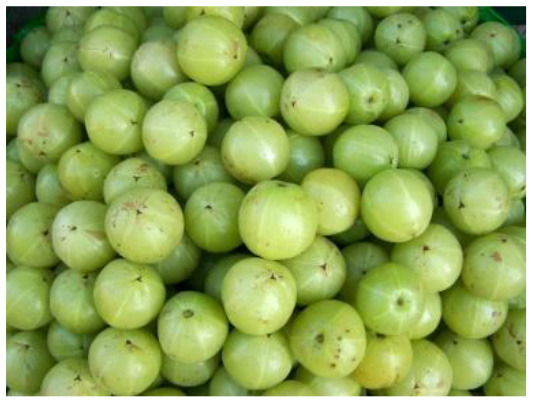	Chlorogenic acid, quercitrin, and myricetin	SARS-CoV-2	Docking simulation using Schrödinger maestro 2018-1 MM share version	–	Bind to NSP15 endoribonuclease, main protease, and receptor binding domain of prefusion spike protein	(27)
	7-ketositosterol, quercetin, epigallocatechin, and phyllaemblic acid-C	COVID-19 M^pro^	GLIDE docking protocol	–	Inhibited the COVID-19 M^pro^ activity by binding to it.	(28)
	–	Bacterial and viral infection	Flow cytometric analysis, and ELISA test	500 mg/kg	Enhanced immunomodulatory efficacy by increasing CD4, CD8, CD16, CD19, IgM, and IgG levels in the blood, and albumin and globulins in serum.	(29)
	Astragalin, catechin, chebulagic acid, corilagin, ellagic acid, gallic acid, geraniin, hypophyllanthin, niranthin, phyllanthin, phyltetralin, and quercetin	Immune-related disorder	–	–	Inhibited the NF-κB signaling pathway; thus, influencing innate and adaptive immunity.	(30)
	Gallic acid, and ellagic acid	Macrophage cell lines, RAW 264.7	MTT assay, reverse transcription-polymerase chain reaction (qRT-PCR), and western blotting assay	2 mg/mL	Down-regulated NF-κB, COX-2, and iNOS.	(31)
	–	Tuberculosis	Nitric Oxide (NO) release assay, and Assay of macrophage phagocytic activity	-	Enhanced proliferation of PBMCs, increased NO release, and improved macrophages phagocytic activity.	(32)
Black Pepper (*Piper nigrum*) 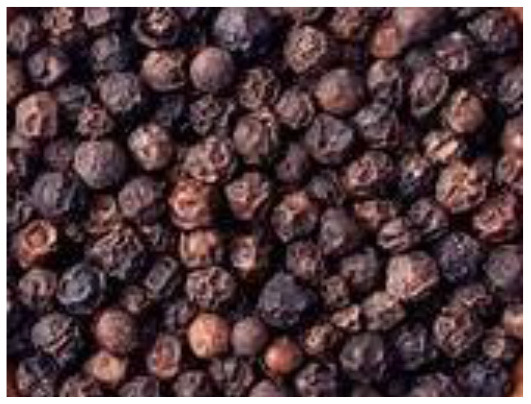	Coumaperine	Bacterial infection	NF-κB-Luciferase Reporter Gene Assay	–	Reduction in transcription Nuclear Factor kappa B (NF-κB) activity.	(33)
	Piperine	Tumor	Cytokine array, and flow cytometry	200 mg/kg	Suppressed some cytokine and chemokine levels including CXCL7, sICAM-1, and L-selectin. Promoted type 1 T helper cell, and suppressed neutrophil, basophil, type 2 T helper cell, and regulatory T cell.	(34)
	Piperine	Food allergy	ELISA test, and RT-qPCR	100 mg/kg	Decreased levels of IgE and mMCP-1. The expressions of Th2 and Th17 cell-associated cytokines were down-regulated and the levels of Treg cell-associated cytokines were up-regulated.	(35)
	Piperine	Upper respiratory tract injury	ELISA test, RT-PCR, and Western blotting analysis	50 mg/kg	Improves the epithelial barrier dysfunction via enhancing the activation of Nrf2/HO-1 signaling	(36)
	Piperine	SARS-CoV-2	Molecular docking	–	Piperine docked to nucleocapsid protein as a potential inhibitor of the RNA-binding site.	(37)
	Piperine	Inflammatory metabolic diseases	Nitrite assay, western blot analysis, and immunofluorescence	50 mg/kg	Inhibited inducible nitric oxide synthase (iNOS)-mediated NO, and IL-1β, IL-6, TNF-α. Suppressed IκB degradation and further inhibited the cytosol-nucleus translocation of the p65 subunit by targeting IKK-β	(38)
	Piperine	–	–	1,000 mg/kg	Enhance the immune function via augmentation of the immunoglobulin (IgM) levels. Increased IgM and IgG levels.	(39)
Cumin (*Cuminum cyminum*) 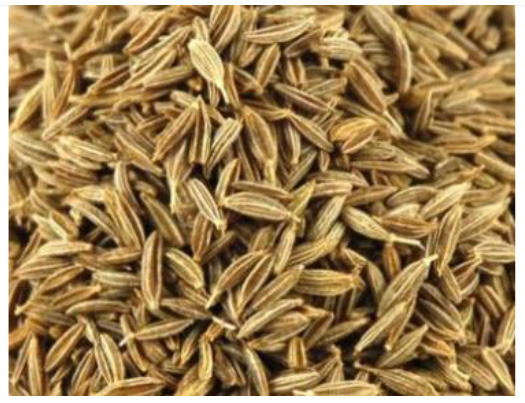	Cuminaldehyde, eugenol	Human campylobacteriosis	Histopathological analyses, *in situ* immunohistochemistry, and pro-inflammatory mediators	200 mg/kg	Alleviated enteropathogenic-induced apoptotic cell responses in colonic epithelia. Secretion of pro-inflammatory mediators, including nitric oxide and IFN-γ to mesenteric lymph nodes.	(40)
	–	–	Immunohistochemical investigations	500 mg/kg	Trk-A immunoreactivity.	(41)
	(3,4,5-trihydroxy-6-((4-isopropylbenzyl) oxy)-tetrahydro-2H-pyran-2-yl) methyl (E)-3-(4-propoxyphenyl) acrylate	Lipopolysaccharide (LPS)-stimulated RAW264.7 cells	Immunoblotting, and ELISA test	–	Suppressed the expression levels of inducible nitric oxide synthase and cyclooxygenase-2. Suppressed the phosphorylation of NF-κB/p65, p-IKK-α/β, and p-IκB.	(42)
	Cuminol, cuminique alcohol, cuminaldehyde, and cymine	Avian influenza (H9N1) and Newcastle disease	Assay of serum antibody titers	250–500 mg/kg	Improved the antibody titers and humoral immune response.	(43)
	Coumarin, and anthraquinone	Bacterial and viral diseases	Enzyme ZellBio Test and alkaline phosphatase (AKP) activity test	10 g/kg	Improved the glutathione peroxidase (GPX) enzyme GPX and AKP activity.	(44)
Garlic (*Allium sativum*) 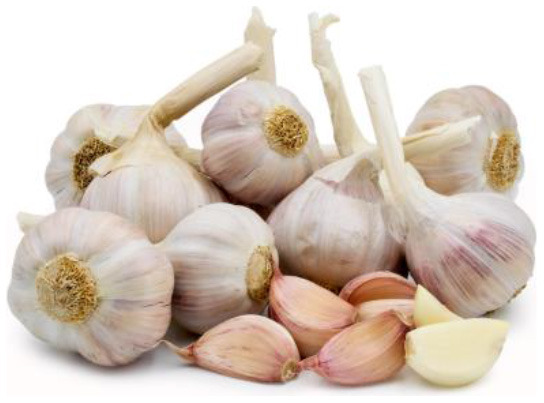	-	ZEN toxicity	Immunity and oxidative stress biomarkers	30 g/kg	Down-regulation of interleukin-4 (IL-4) and interleukin 1 beta (IL-1β) genes alongside significant up-regulation of tumor necrosis factor-alpha (TNF-α) and heat shock protein 70 (HSP70) genes.	(45)
	Sulfur-containing compound	–	Hematological profile	10 g/kg	Improved total leucocyte count (TLC), lymphocytes, and monocyte along with neutrophil count which ultimately strengthened the innate and adaptive immunity.	(46)
	Allicin	Copper sulfate-induced toxicity	Hematological and immunological parameters test	10 g/kg	RBCs, Hb, PCV%, MCV, WBCs were increased and by modulating the deranged differential leukocyte count and phagocytic activity as well as a serum level of nitric oxide, lysozyme activity, and IgM.	(47)
	Allicin, alliin	–	Commercial test kit	0.25–0.075 g/kg	Improved immunoglobulin M (IgM), and IgG.	(48)
	Allicin	Infectious diseases	Immunological parameters test	–	Improved superoxide dismutase (SOD) and catalase (CAT) activities.	(49)
	–	COVID-19	–	–	Decreased the expression of proinflammatory cytokines and reverse the immunological abnormalities.	(50)
Giloy (*Tinospora cordifolia*) 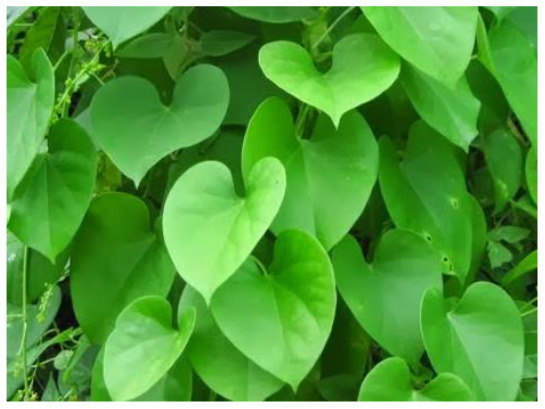	20a hydroxy ecdysone, amritoside, apigen-6-C-glucosyl7-O-glucoside, epicatechin, and tinosporine B,	COVID-19 M^pro^	GLIDE docking protocol	–	Inhibit the COVID-19 M^pro^ activity by binding to it	(28)
	Cordifolioside-A, Magnoflorine, β-Ecdysone, and Palmatine	SARS-CoV-2 spike-protein	Cytological study	142 μg/kg	Reduced lipopolysaccharide (LPS) induced expression of TNF-α, IL-6, and IL-1β.	(51)
	Berberine	COVID-19 3CL^pro^	Molecular docking analysis	-	Inhibit viral replication by binding to the active site.	(52)
Ginger (*Zingiber officinale*) 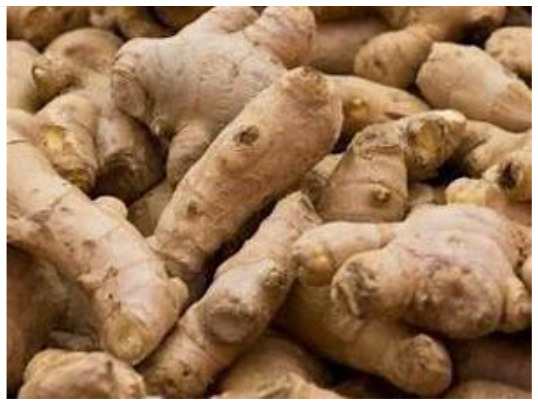	Gingerols	–	Hematological profile	10 g/kg	Improved total leucocyte count (TLC), lymphocytes, and monocyte along with neutrophil count which ultimately strengthened the innate and adaptive immunity.	(46)
	Zingiberene	*Aeromonas hydrophila* infection	Immunological assay	10 g/kg	By increasing the production of H_2_O_2_ and NO and SOD and lysozyme activities.	(53)
	–	*Aeromonas hydrophila* infection	Plasma humoral immune analysis	10 g/kg	Lowered the expression of tumor necrosis alpha (tnfa), interleukin 1 beta (il1b), and interleukin 8 (il8), and higher interleukin 10 (il10) genes.	(54)
	Zingiberene, gingerol, and zingerone	–	Mucosal parameters test	1–4 g/kg	Increased mucosal immune parameters such as lysozyme, alkaline phosphatase, and total Immunoglobulins.	(55)
	Gingerol, gingerdiol, and gingerdione	Bacterial diseases	RT-PCR	30 g/kg	Lysozyme mRNA expression levels were up-regulated.	(56)
	Gingerol, shogaols, gingerdiol, and gingerdione	Bacterial diseases (*Escherichia coli*)	qRT-PCR	15 g/kg	Regulating the IL-1β, IL-6, and IFN-γ cytokines expression levels.	(57)
Tulsi (*Ocimum tenuiflorum*) 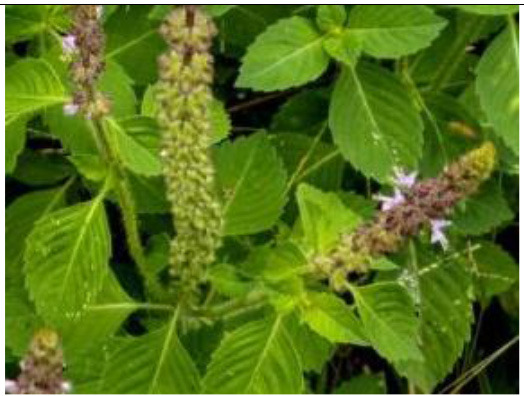	–	Asthma	Immunologic and inflammatory markers measurement	0.75, 1.50, and 3.00 mg/mL	By increasing the IL-4, IgE, PLA2, and TP levels, and decreasing the IFN-γ/IL-4 ratio.	(58)
	–	*Staphylococcus aureus* and *Escherichia coli*	RT-PCR	5–10 g/kg	Improved the relative mRNA expression levels of TLR 2, TLR 4, and TLR 7.	(59)

*- Not reported*.

**Figure 2:**
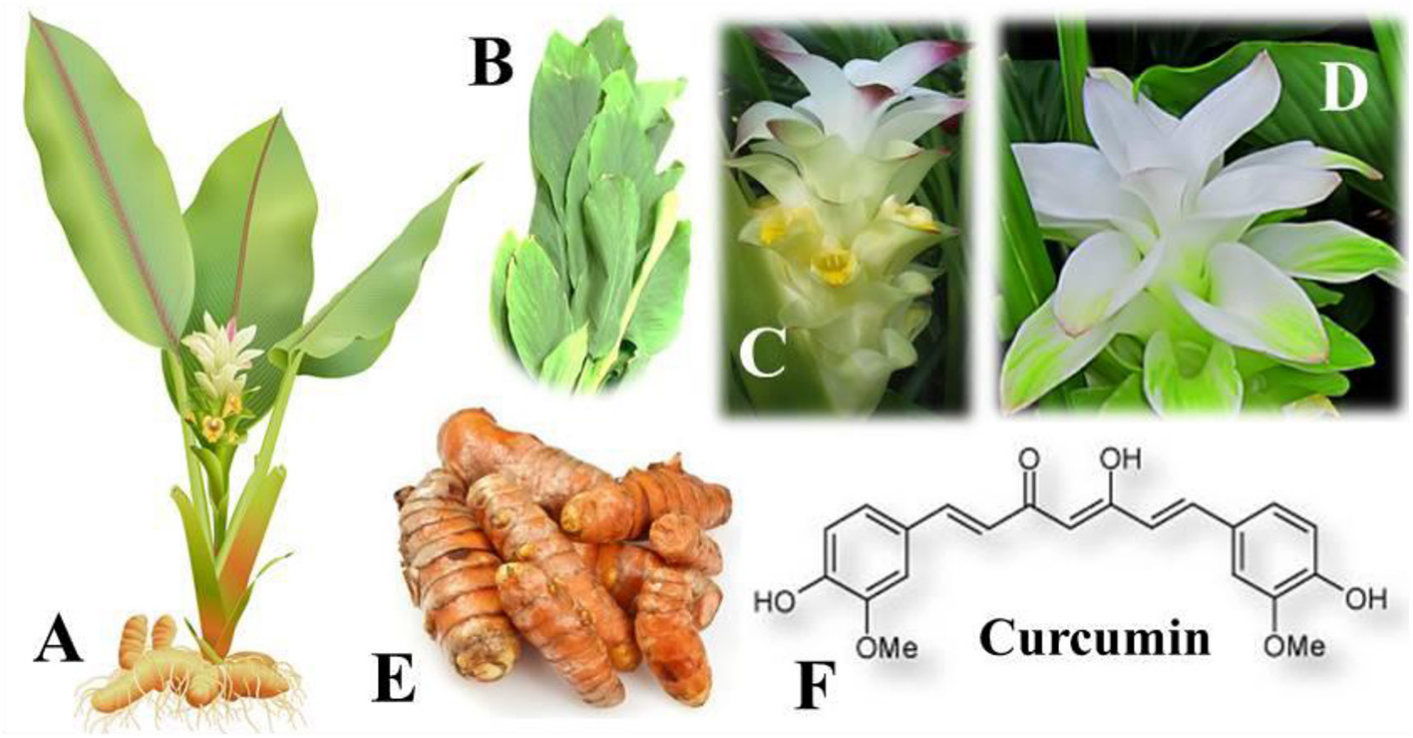
Turmeric (*Curcuma longa* L.) plants are with the following features: **(A)** A highly branched standing *C. longa* plant with cylindrical rhizomes of yellowish to orange color. **(B)** Broad long and simple leaves with long petioles (leaf stems) grow from branching rhizomes that lie just below the surface of the soil. **(C)** Inflorescence is terminal, spike-shaped, and cylindrical, having laterally green united bracts with reddish spots. **(D)** It produces very pretty, tall white flower spikes. **(E)** *C. longa* rhizomes with yellowish to orange color. **(F)** The natural and major bioactive compound of the *C. longa* plant.

**Figure 3:**
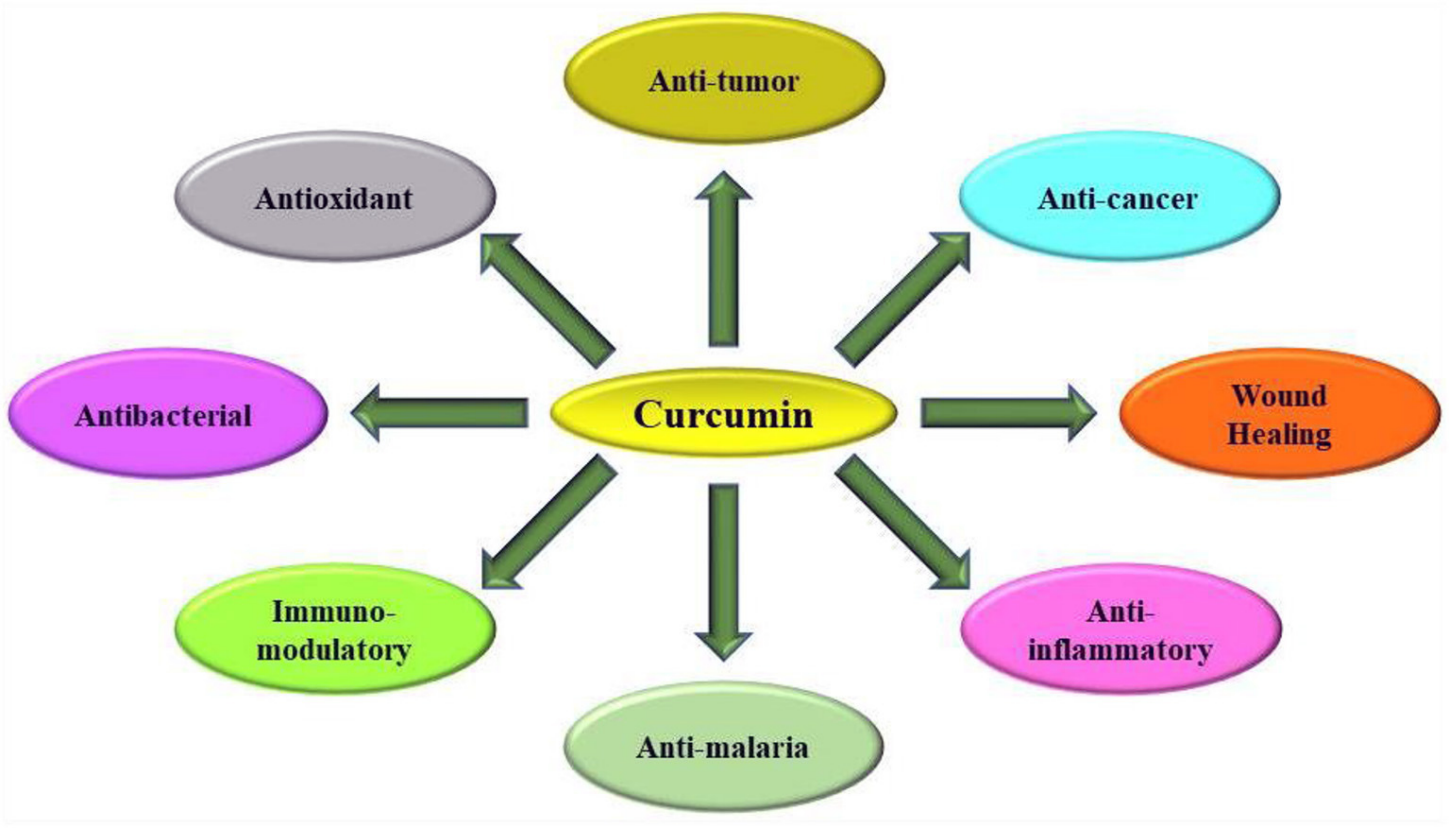
Important biological activities performed by curcumin in human health (3, 65).

Among many bioactive compounds, curcumin, a natural and major bioactive compound found in rhizomes of turmeric (*Curcuma longa* L.) plant (Figure 2) which belongs to the family, Zingiberaceae. It is a hydrophobic phenolic compound with the chemical name 1,7-bis (4-hydroxy-3-methoxyphenyl)-1,6-heptadiene-3,5-dione. There have been 3 major compounds, including curcumin (diferuloylmethane), demethoxycurcumin, and bisdemethoxycurcumin, which are chemically part of the “curcuminoid” family. Curcumin is the most biologically active form of them. The native Indian plant is currently farmed in Asian countries, such as Indonesia and China that are characterized by warm and wet tropical climates. Apart from its uses as a spice or flavoring or coloring agent in food preparations from centuries, it is commonly used to cure various viral infections such as several cases of flu and cold symptoms (65). Curcumin from plant materials is obtained using several methodologies, from traditional extraction processes, like Soxhlet extraction, maceration, and solvent extraction to recent extraction technologies, such as extraction by means of ultrasound, microwaves, enzymes, and supercritical liquids. The isolation and purification of curcumin from crude extracts is accomplished by techniques such as column chromatography, high-performance liquid chromatography (HPLC), high-speed counter-current chromatography, supercritical fluid chromatography, either alone or in combination (66–68). The biological effect of curcumin on some of the global ailments, including cardiovascular diseases, diabetes, metabolic syndrome, and arthritis, is defined by molecular targets and physiological effects on animals (69). Multiple mechanisms have been derived from these molecules to protect the health, owing to its biological properties including immuno-modulatory or immunity-boosting characteristics (Figure 3) (3, 65).

Pure curcumin is a yellow-orange-colored crystalline substance, which normally comes in a powder form. However, the direct application of curcumin is much less due to a slightly bitter taste, poor water solubility, poor chemical stability, particularly under alkaline conditions, and low bioavailability, attributed to low bioaccessibility and chemical transformation due to metabolic enzymes in the gastrointestinal (GI) tract. Further, during storage curcumin is more susceptible to chemical degradation, especially when exposed to high temperatures, alkaline conditions and light (70, 71). It is essential to overcome these barriers by developing efficient approaches so that curcumin can be successfully incorporated into functional foods, supplements, and pharmaceuticals. Therefore, many researchers are investigating various methods to conquer such hurdles and among that encapsulation technology is found to be one of the most significant means of improving the bioavailability, water solubility, and protecting curcumin against chemical degradation (72, 73). Both microencapsulation and nanoencapsulation have been found to be efficient processes to overcome the above problems. These techniques can even mask the bitter flavor of curcumin when consumed directly or in a food product.

Further, curcumin is safe for consumption, even at relatively higher levels as per toxicity studies. Because of its broad-spectrum biological activity and low toxicity, it has been extensively researched as a nutraceutical component for use in functional foodstuffs (73). Such curcumin-incorporated food products will improve the immunity of the human body and may combat the viral infections including coronavirus. However, an essential step toward functional development is the investigation of the biological effects and immune-enhancement properties of curcumin in the healthy population and people diagnosed with non-compatible diseases by giving greater information about its therapeutic advantages (with respect to *in-vitro* and animal studies). Therefore, the selection of optimal extraction conditions to obtain extracts and the biological activity of the extracts play a crucial role in recognizing and using curcumin in the manufacture of functional foods (74).

Taking into account the above aspects, the effective extraction and purification techniques which should be safe, eco-friendly, economical and efficient, must be followed to get pure curcumin. Therefore, the review addressed the different methods of curcumin extraction, isolation, and quantification in order to give the researchers a better understanding of the processes. The objective of this review is to assess the immunomodulatory impact of curcumin on the novel coronavirus and its potential application in the prevention of COVID-19. It also focuses on the use of curcumin to produce functional foods that can improve human body immunity to novel coronavirus. The main purpose of the review is therefore to address curcumin's immunological activity that will help food scientists and researchers formulate functional food to minimize the outbreak of the coronavirus.

## Curcumin Associated Functional Foods, Human Immune System, and COVID-19

The use of food supplements like curcumin may be important if the immune system is to be strengthened and disease conditions like respiratory infections are to be prevented (3, 4, 75–78). It can provide a promising option in terms of functional food products and is an important part for elderly patients or people at risk, whether in hospitals or nursing homes. At every stage of human growth and development, the proper intake of essential nutrients is needed, particularly in certain physiological conditions (old age, severe shortcomings in nutrition, and pathophysiologically stressful situations). The natural bioactive compounds like curcumin extracted from plants have become important in human health nowadays, leading to food scientist's application into food and functional food formulation (3, 4, 22–24, 77, 79). Although the development of COVID-19 vaccines is considered an optimal choice toward the prevention of the disease, their development requires an expensive and time-consuming process besides evaluation of toxicity and effectiveness in the population (4, 80–84). Therefore, it may be vital to determine the impact of certain food ingredients as possible candidates for management with a targeted approach. The use of curcumin has been demonstrated in traditional herbal medicine (2, 4). Curcumin possesses a wide variety of biological activities, improving human health, as illustrated in Figure 3, which has piqued the interest of many researchers and scientists (75, 81, 85–89). In addition to these functions, immunological activity is the most essential property of curcumin and it has therefore been shown to be used against anti-immune diseases (77, 90–92).

**Figure 4:**
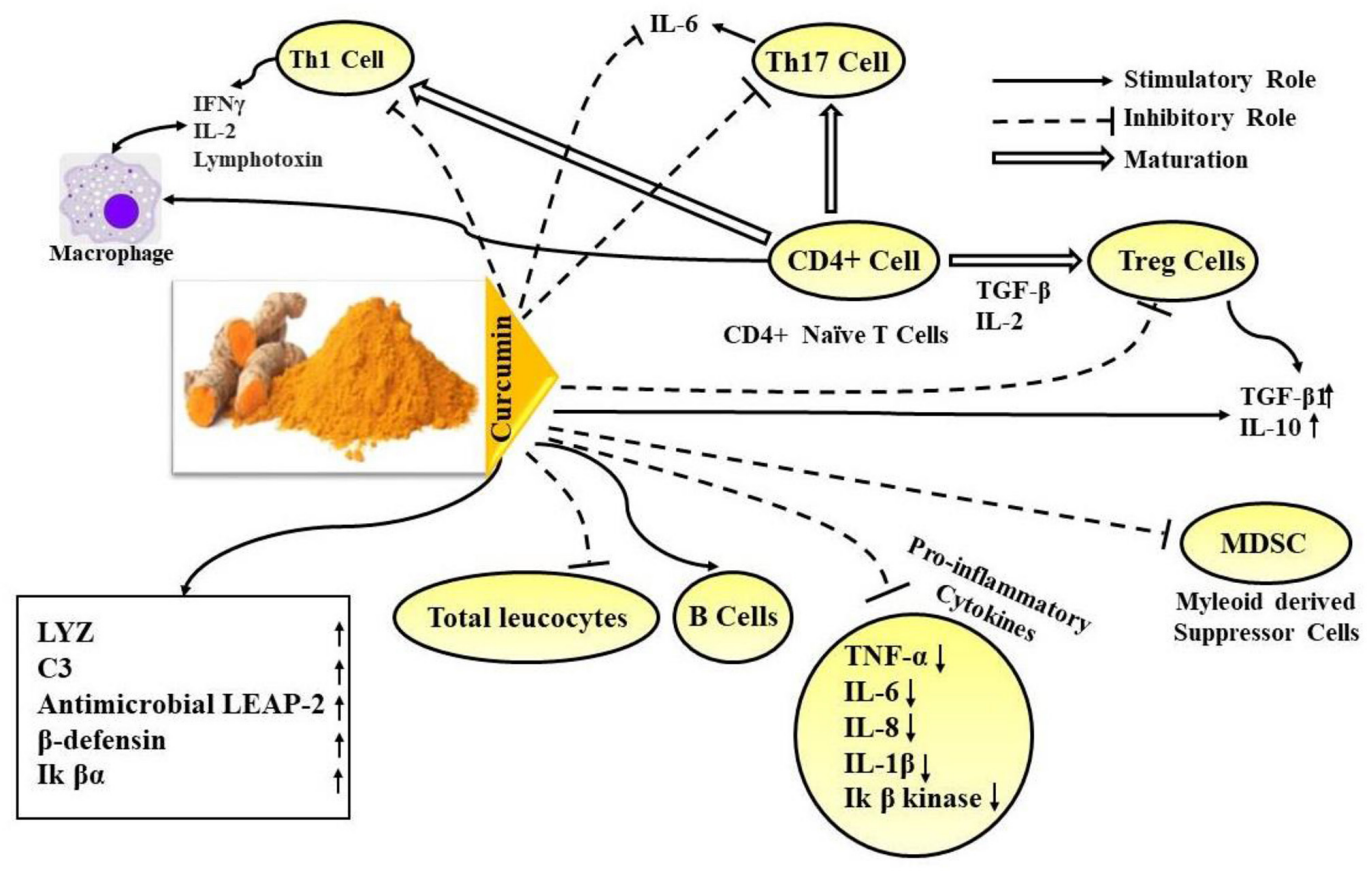
Schematic presentation on immunomodulatory effects of curcumin. Naïve CD4+ T cells are capable of differentiating into different T cell subsets like the Th1, Th2, Th17, Treg cells, etc. The possible effects of curcumin on some of these specific types of T cells that differentiate out of the naïve T cells have been shown here. Curcumin is seen to exert an inhibitory role in Th1 and Th17 cells which are known to be key players in pro-inflammatory T cell-mediated responses. It has also been found to inhibit Treg, Myeloid-derived suppressor T cells, overall total leucocyte counts. Curcumin has also been found to inhibit pro-inflammatory cytokine response like production of TNF-α, IL-6, IL-8, IL-1β, and Ik β kinase and promote the upregulation of anti-inflammatory cytokines like IL10, TGF-β1. Curcumin has also been seen to increase B-cells and the production of immunoglobulins like IgA and IgG.

Recently, some studies have identified potential molecular targets in the viral replication cycle to determine the potential role of curcumin in the fight against SARS-CoV-2 (4, 84, 93–98). White blood cells (WBCs) are the key players in human immune systems and specifically lymphocytes have been found to produce increased levels of immunoglobulins (IgG and IgM) when Nawab et al. (77) investigated the effect of curcumin on the immune profiles of the blood. Many researchers have worked on curcumin and reported the effects of curcumin on immunity as shown in Table 2. The immunomodulatory effects of curcumin with a specific focus on the possible effects on the different types of WBCs have been described in Figure 4. The image shows the overall effects of curcumin on different types of WBCs (especially the different types of T cells).

**Table 2:** Some recent findings on the effect of Curcumin on the immune system.

**Bioassay**	**Dose**	**Remark**	**References**
ELISA assay	100–200 mg/kg diet	Significant increase in estradiol, follicle-stimulating hormone levels, IgA, IgG, luteinizing hormone, and complement C3 activity in the serum (*P* < 0.05).	(99)
Flow cytometry	-	Curcumin suppressed inflammatory monocytes across the blood-brain barrier (BBB) in Experimental Autoimmune Encephalomyelitis (EAE) mice, suppressed the spread of microglia, and limited infiltration of other effector immune cells, resulting in a reduction in EAE morbidity from 100 to 30%. It was due to the immunomodulatory impact of curcumin-loaded high-density lipoprotein-mimicking peptide-phospholipid scaffold (Cur-HPPS) on inflammatory monocytes, which inhibited the activation of NF-κB and decreased the expression of adhesion-and migration-related molecules.	(88)
Serum biochemical parameters assay	196.11–788.52 mg/kg diet	Curcumin up-regulated the mRNA levels of LYZ, C3, and antimicrobial peptides [hepcidin, liver-expressed antimicrobial peptide-2 (LEAP-2), β-defensin]; anti-inflammatory cytokines of interleukin-10 (IL-10); an inhibitor of κBα (IκBα); and transforming growth factor β1 (TGF-β1); whereas, down-regulated pro-inflammatory cytokines of tumor necrosis factor-α (TNF-α), IL-6, IL-8, and IL-1β; IκB kinases (IKKα, IKKβ, and IKKγ) and nuclear factor kappa B p65 (NF-κB p65) mRNA levels in the liver and blood.	(89)
Serum biochemistry assay	100–200 mg/kg diet	A substantial reduction in total leukocytes as a result of the reduction in lymphocytes was observed in animals receiving curcumin and was observed for total serum protein and globulin levels.	(75)
Serum inflammatory cytokines analysis	100-300 mg/kg diet	The curcumin treatment group had reduced inflammatory responses (TNF-α, IL-1β, and IL-6,) as compared to the control group.	(92)
Western blot analysis	100–300 mg/kg diet	TLR4, PCNA, and its downstream gene expression, as well as protein expression (NFκB, TLR4, and PCNA), were significantly downregulated in the heat stress curcumin supplemented group as compared to the control group.	(92)
ELISA assay	200 μg/mouse	Curcumin suppressed the development of antigen-specific IgE and IgG1, inhibited CD4+ T function, and decreased ovalbumin-sensitized B-cell memory.	(100)
Immunofluorescence assay	-	Expression of *p*-STAT3Y705 and PD-L1 was similarly decreased *in vivo*.	(101)
Flow cytometry	-	After curcumin treatment, the anti-tumor immune response was remarkably improved by rising CD8 positive T cells and decreasing Tregs and MDSCs.	(101)
Hemagglutination assay	5–10 mg/kg diet	Curcumin nanoparticle significantly induced primary humoral immune response with 9.00 ± 1.00 antibody titer (*P* < 0.05), free curcumin suppressed immunity with 3.33 ± 0.67 antibody titer compared to control. Similar findings were found with secondary humoral antibody titers.	(102)
Intracellular staining	10,000 mg/kg diet	Curcumin diet reduced all populations of Th1/Th2/Th17 cells and attenuated various symptoms such as splenomegaly in scurf mice.	(91)
Cytokine measurement assay	10,000 mg/kg diet	*In vitro* studies showed that curcumin treatment directly decreased the development of Th1/Th2 /Th17 cytokines in CD4 + T cells from IL-4, IL-17A, and IFN-γ.	(91)
Oxidative stress and immunological assay	50–200 mg/kg diet	Total IgM and IgG levels increased significantly, in particular.	(85)

The key pathway of entry for SARS-CoV-2 is through our respiratory system, and it has been particularly found to enter cells by binding to the ACE2 receptor (angiotensin-converting enzyme 2) (78). The spike protein present on the COVID-19 surface is pinched within the host cell that binds to the ACE2 receptor (103). TMPRSS-2 is another active site for the entry of this coronavirus (104). Curcumin has been found to have a stronger binding capacity to the ACE2 receptor, and thus may potentially block the entry of SARS-CoV-2 (105). It also has been seen to decrease the expression of TMPRSS-2, which demonstrated the ability of curcumin against SARS-CoV-2 (87).

**Figure 5:**
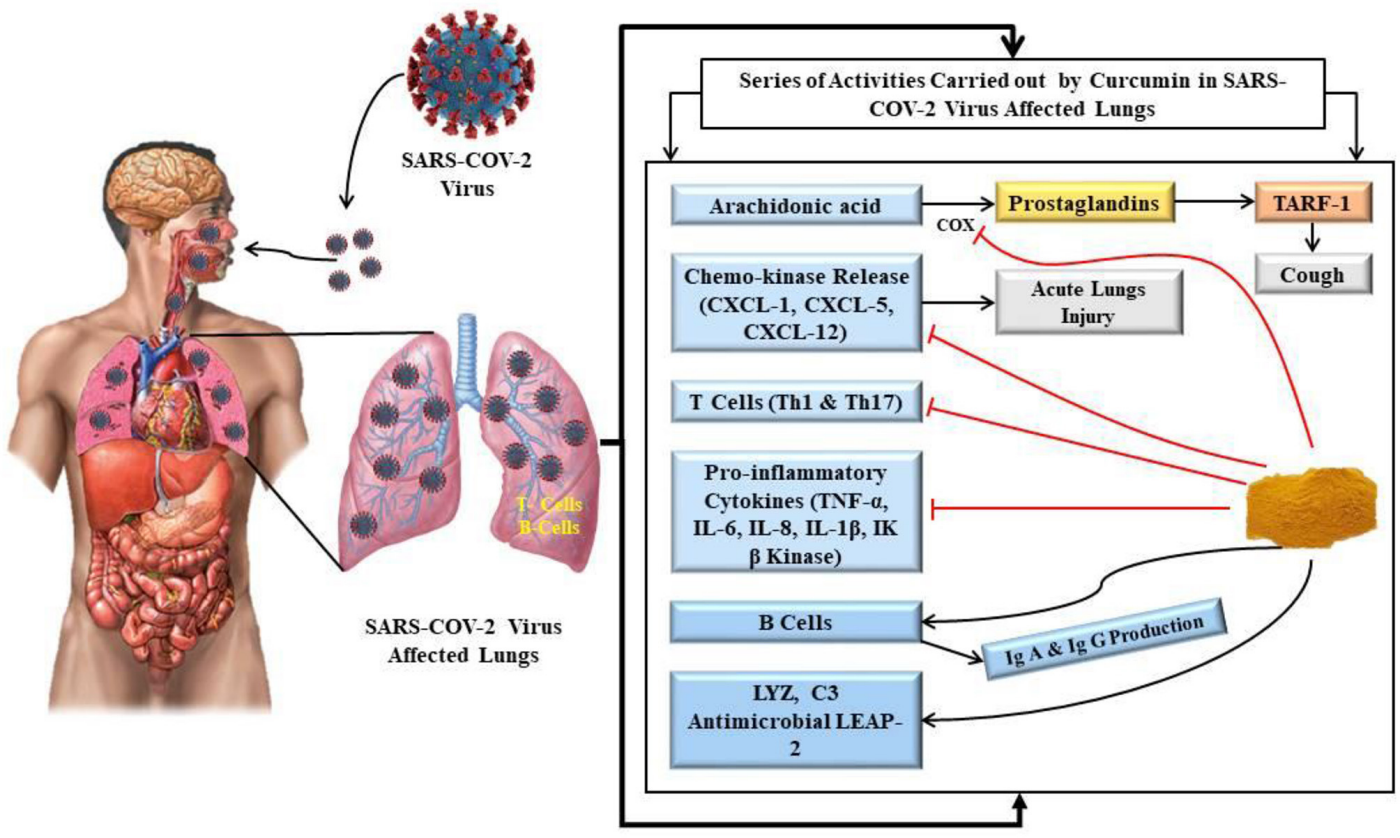
Schematic presentation on molecular mechanisms describing effects of curcumin on lungs infected with SARS-COV-2 virus.

As mentioned above, the SARS-CoV-2 virus enters the human body through the upper respiratory tract. While the majority of the patients are seen to manifest mild to moderate symptoms, some are seen to have a severe pathological immune response requiring hospitalization, life support and may eventually turn out to be fatal. Many patients have the typical symptoms of upper respiratory tract infection like cough, sore throat, running nose, fever and others (106–111). Patients with severe respiratory illness are seen to have hyper-immune response diffuse alveolar damage, necrosis of the epithelium, epithelial necrosis, fibrin deposition and hyaline membrane formation. At the cellular and molecular level, a poor neutrophil to lymphocyte ratio is considered a poor prognostic indicator for COVID-19 (112, 113). An increased pro-inflammatory response may be an eventual harbinger of a cytokine storm. Curcumin has been found to have beneficial effects on many of these pathological processes (3, 4, 15, 16, 68, 93, 111, 114, 115). Curcumin can reduce cough induced by bradykinin (115, 116). It has an inhibitory effect on chemokine release and thus may prevent acute lung injury (84, 94, 115, 117). The inhibitory role of curcumin on Th1 and Th17 cells is also expected to have a beneficial role. Besides these, as has been mentioned earlier, curcumin has an inhibitory role on the production of pro-inflammatory cytokines like IL-6, IL-8, IL-1 β, Ik β kinase etc. The stimulatory role of curcumin on B cells may be beneficial in antibody production. It is also expected to prevent an eventual acute respiratory distress syndrome. A schematic diagram with the possible mechanisms of curcumin action in COVID-19 has been shown in Figure 5. While it is difficult to confirm the optimal dosage of curcumin in COVID-19 (owing to the paucity of data available), it is important to note that curcumin supplementation is considered relatively safe (3, 4, 10, 15, 16, 110, 111, 115, 116) and well-tolerated even at high doses up to 8 g/day as shown in different clinical trials (118). Generally, curcumin doses in the range of 0.5–1.5 g/day are seen to be helpful in clinical improvement in conditions like inflammatory conditions, and hence such a dose may be explored in COVID-19 as well (118).

**Figure 6:**
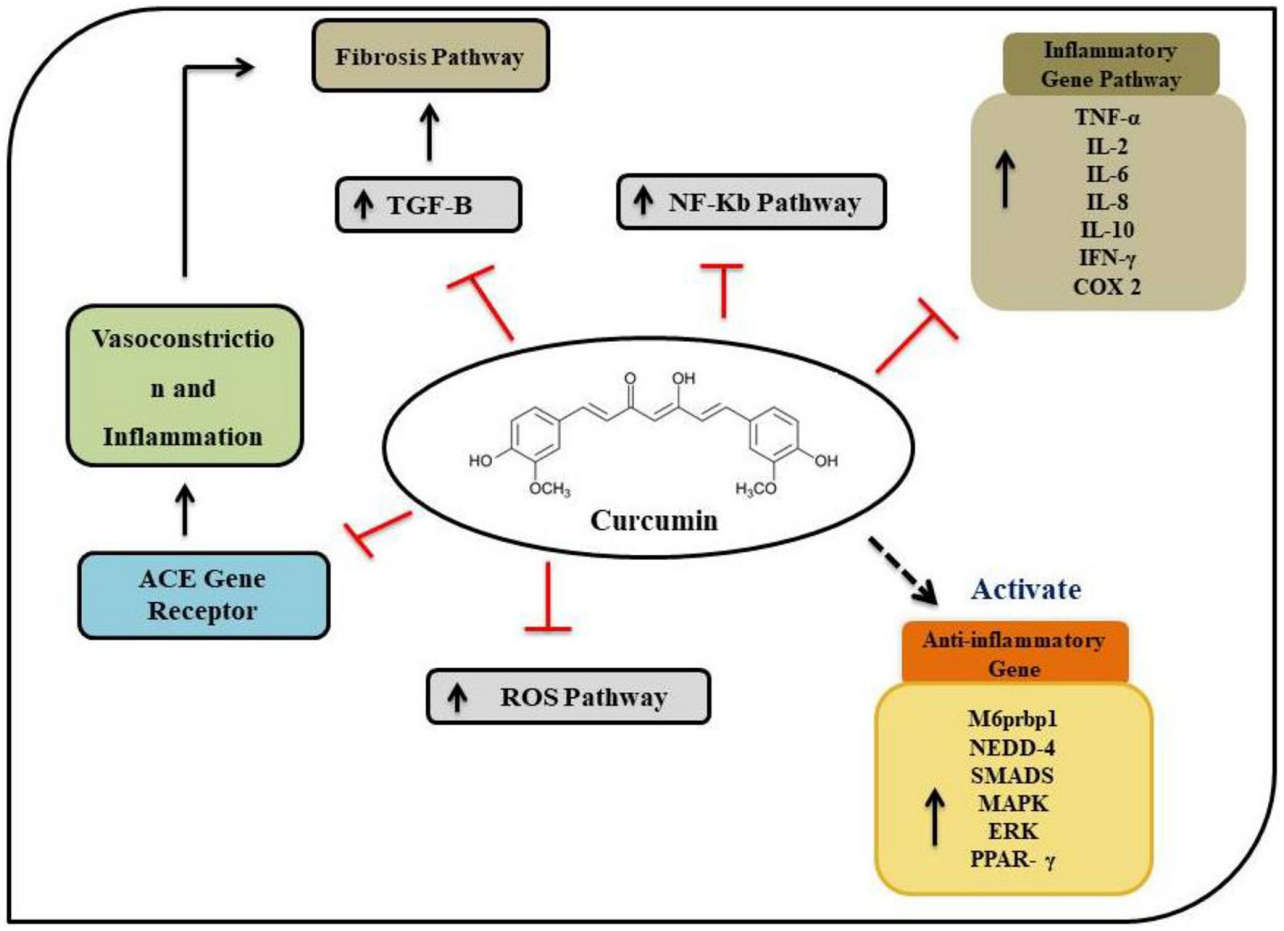
Potential molecular targets for curcumin in the different gene receptors and block the SARS-CoV-2 replication signaling pathway.

Recently, an *in-silico* molecular-docking study of curcumin has found a strong potential antagonist to the human ACE2 receptor and the SARS-CoV-2 spike S protein (119). In addition to these curcumins, virus replication can also be prevented by reducing the number of plaques (15). Curcumin is known to be a potent proteasome inhibitor that raises the p-53 level, and the epigenetic regulatory function of curcumin against viruses arises from its ability to interact with various biological targets to trigger molecular signaling pathways, such as apoptosis and inflammation (120). The future goal of curcumin to accomplish potential molecular targets in the different gene receptor to block replication of SARS-CoV-2 has been shown in Figure 6.

**Figure 7:**
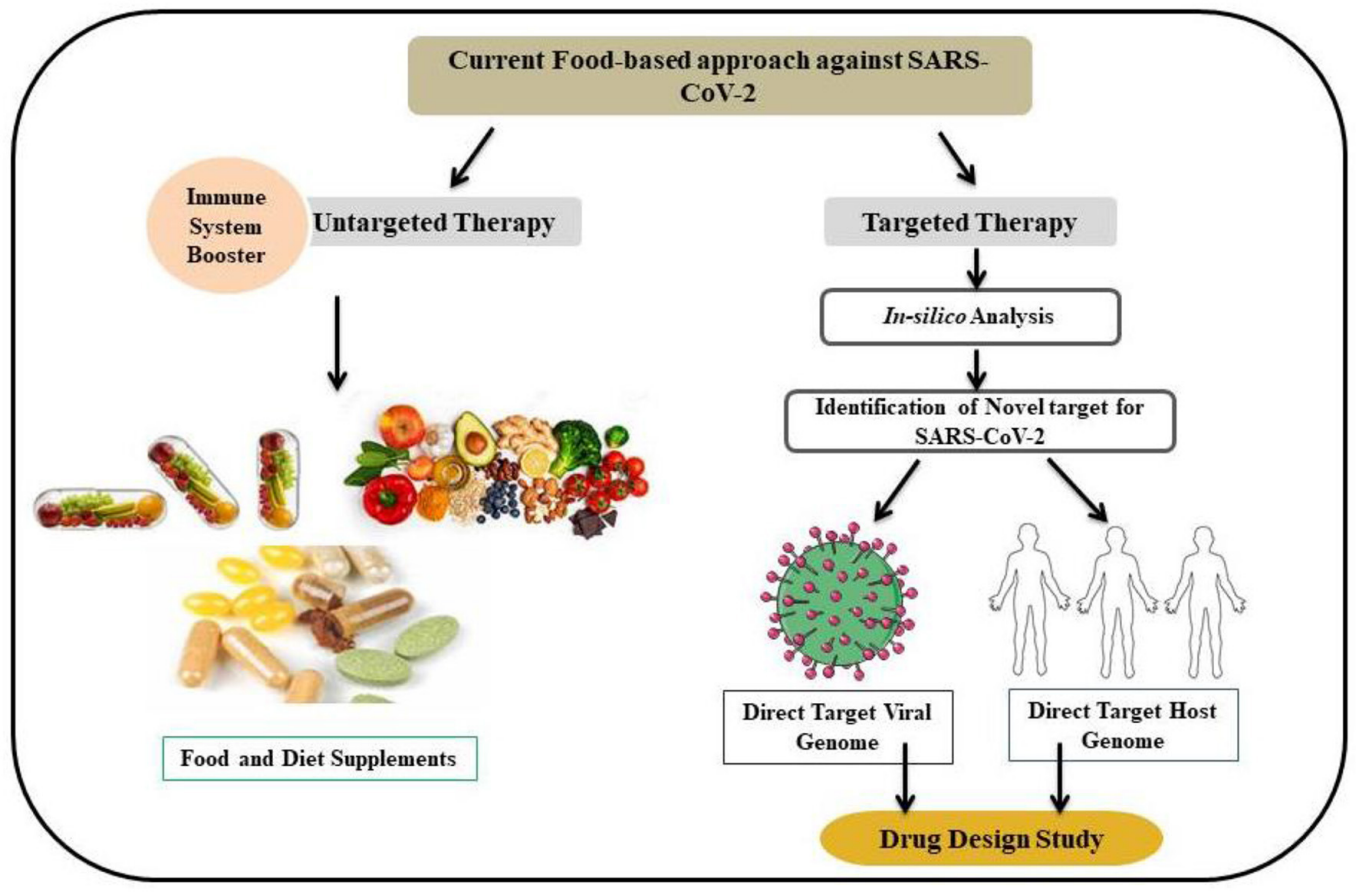
A pattern of the current approach to research in food components and diets used against SARS-CoV-2. The untargeted approach is focused on improving the immune system by food nutrients and the targeted approach focuses on the interaction of protein compounds with the host and virus systems.

In severe cases of COVID-19, acute respiratory distress syndrome (ARDS) is seen to occur due to the release of a large number of pro-inflammatory cytokines. Curcumin can potentially reduce the pro-inflammatory cytokines such as interleukin-6 (IL-6), IL-1β, IL-4, tumor necrosis factor-α (TNF-α), and MCP-1 by blocking the nuclear factor-κB (NF-κB) and TNF-α (16). Collective information on the impact of curcumin on the human immune system at different levels and the possible efficacy of curcumin against COVID-19 has been addressed in this section (Table 3). Therefore, their incorporation into the food system to develop functional foods can be a novel method of providing adequate curcumin to the human body so that an individual can fight against coronavirus during or before the case. The detailed approach has been shown in Figure 7. In an open-label non-randomized clinical trial, Saber-Moghaddam et al. (110) revealed that oral nano-curcumin formulation was efficient in managing coronavirus infection in patients. It was observed that major symptoms including cough, fever and chills, tachypnea, and myalgia resolved significantly faster in the curcumin group. Similar results were also noticed by other researchers (Table 3).

**Table 3:** Various *in-vivo* studies conducted in humans against the Covid-19 virus.

**Number of patients included in the trial**	**Age (year) of patients**	**Male/female ratio**	**Location of trial**	**Treatment protocols followed during the treatment**	**Major finding(s)**	**References**
60	**–**	**–**	Mashhad, Iran	Sinacurcumin soft gel containing 40 mg curcuminoids as nanomicelles, two soft gels twice a day	A significant difference was observed in the curcumin-treated group for symptoms like cough, chills, and disturbances in smell and taste. The lymphocyte count was significantly higher while CRP serum level was lower in the treatment group at the end of 2 weeks.	(116)
99	39–70	67/32	Wuhan, China	Oxygen therapy, antibiotic treatment, Antifungal and antiviral treatment, Glucocorticoids and intravenous immunoglobulin therapy	Older males with comorbidities were found to be more affected by the virus; which can lead to severe or in some cases even fatal respiratory infections like acute respiratory distress syndrome.	(121)
145	47.5 (Average)	79/66	Zhejiang, Chin	Antiviral and corticosteroid therapy, respiratory support	It was noticed that older patients with comorbidities like diabetes mellitus or obesity were more prone to have a severe condition	(106)
> 100	–	–	Wuhan, Jingzhou, China	Treatment with chloroquine phosphate	Treatment with the compound resulted in the prevention of infection with the virus; it was interfering with the glycosylation of cellular receptors of SARS-CoV-2.	(122)
20	> 12	–	Marseille, France	Treatment with hydroxychloroquine (600 mg/day) and azithromycin	As compared to controls, there was a reduction of the viral carriage at D6-post inclusion suggesting that hydroxychloroquine is highly efficient in reducing viral load and further its effect is reinforced by azithromycin.	(123)
41	25–64	30/11	Wuhan, China	Oxygen support, antibiotics*, antiviral (oseltamivir; 75 mg twice daily), corticosteroid (methylprednisolone), renal replacement therapy	The Covid-19 infection caused clusters of severe respiratory illness similar to severe acute respiratory syndrome coronavirus and was associated with ICU admission and high mortality.	(107)
155	42–66	86/69	Wuhan, China	Oxygen support, corticosteroid*, treatment with expectorant, antiviral: arbidol, lopinavir, ritonavir, interferon inhalation, and immune enhancer (thymalfasin, immunoglobulin)	It was observed that older males with the refractory disease were more prone to infection with the virus.	(124)
21	18–57	5/16	Mashhad, Iran	Treated with Sinacurcumin^®^ soft gel (two capsules twice a day containing 40 mg curcuminoids as nanomicelles)	Compared to the control group, the treatment of curcumin nanoformulation fastened the resolution time of COVID-19-induced symptoms, improved oxygenation, and also reduced hospital stay time.	(110)
120	–	–	Mashhad, Iran	Nanocurcumin (160 mg/day for 21 days)	Compared to the placebo group, in mild and severe COVID-19 patients who received nanocurcumin, a significant reduction in Th17 cells and Th17 cell-related cytokines levels were found.	(111)
135	36–55	72/63	Northeast Chongqing, China	Oxygen support, treatment with antibiotics*, antiviral agents (Kaletra), corticosteroid*, traditional Chinese medicine (TCM) therapy, and renal replacement therapy	It was revealed that antiviral agent Kaletra, as well as TCM, could play a significant role in viral pneumonia treatment.	(108)
69	35–62	32/37	Wuhan, China	Oxygen support, treatment with antibiotics*, antiviral*, corticosteroid*, antifungal*, arbidol, moxifloxacin, plus interferon therapy*	It was observed that more deaths occurred in the SpO_2_ < 90% group. The patients of this group were older with comorbidities. They had a higher plasma level of lactate dehydrogenase, C reactive protein, interleukin (IL) 6, and IL10 than the SpO_2_ ≥ 90% group.	(125)
200	55 (Average)	98/102	Yichang, Hubei Province, China	Medical treatment, respiratory support, and renal replacement therapy	The COVID-19 infection was of clustering onset; It was resulted in the cause of severe respiratory disease and in few cases even death.	(109)

* *Doses were not reported*.

## Extraction, Isolation and Quantification of Curcumin

Various processes have been followed in order to increase the availability of bioactive compounds, such as the selection of plant material and its component, cleaning, and subsequent drying followed by extraction and purification of the desired compound (126, 127). The extraction method can be categorized as traditional and modern extraction techniques. The most common traditional extraction methods are Soxhlet extraction (66, 127–129) and maceration (130, 131). Whereas, ultrasound-assisted extraction (UAE) (132, 133), microwave-assisted extraction (MAE) (134), enzyme-assisted extraction (128) are the most common methods in modern extraction process. Despite many drawbacks such as high temperatures, high operating times, and high organic solvent use, the traditional extraction method is commonly used due to its simple procedures and low operating costs (127, 135, 136). Identification and quantification of bioactive compounds are accompanied by the use of one or more chromatography techniques such as thin-layer chromatography (TLC), high-performance liquid chromatography (HPLC), and ultra-HPLC with a mass spectrometer (MS) (66, 136–140).

**Figure 8:**
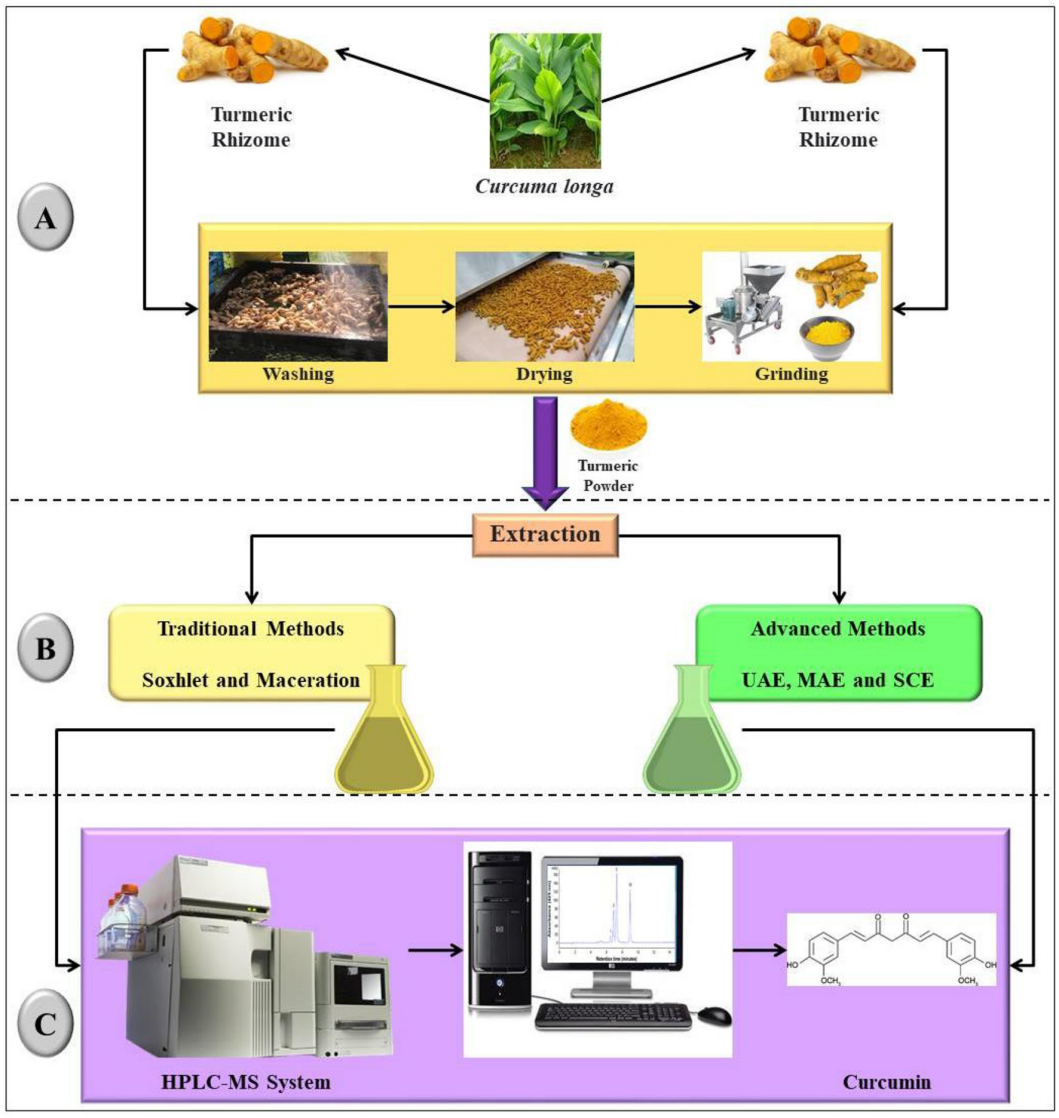
Schematic presentation of extraction and isolation of curcumin with different extraction techniques coupled with an analytical technique for identification and quantification. **(A)** Sample Preparation: In this step, turmeric rhizomes were collected and appropriately washed. Then drying and grinding were done. Drying and size reduction are essential for processing, as size plays an important role (smaller the size, higher the diffusion of bioactive compounds from source to solvent) in the extraction process (136, 141, 142). **(B)** Extraction and Purification: The ground samples are then subject to an extraction procedure. The most commonly used extraction process for curcumin is the traditional method due to its low cost of operation and simple handling. Among the different extraction processes, the reflux method of powdered turmeric with dichloromethane achieved higher extraction yields ranging from 81.81 to 86.36% (138). However, as a green extraction technology, the subcritical water extraction method also raises extraction yields to 76% compared to other modern extraction techniques (137, 143). **(C)** Identification and Quantification: The most precise technique used to characterize curcumin was HPLC equipped with a column C18 of several lengths (100–250 mm), inner diameters (2.1–4.6 mm), and particle sizes (0.45–5 μm) (128, 137, 139, 140). The HPLC is an advanced method of liquid chromatography with a high separation capability. Further, the samples were scanned at different modes. Comparing the observed MS/MS spectra with those found in the literature was the primary tool for identifying the bioactive compounds.

Sample preparation is a basic step followed prior to the extraction of the desired bioactive compound. The primary step is the selection of the proper plant variety and the part of the plant that will produce the targeted phytochemical (136, 141). In our case, therefore, the plant is turmeric and the rhizome of the turmeric plant has been used to extract curcumin (Figure 8A). The findings show that all researchers had extracted the powdered sample as it increases the extraction yield (Table 4). Yulianto et al. (145) obtained the higher extraction yield on using high temperature (140 and 150°C) for extraction and a lower solid:liquid ratio (1:10) also offered a higher concentration of the curcumin. Apart from these, it is also important to choose the correct solvent, as it affects the extraction yield as well as increases the toxicity of the extract. Shirsath et al. (74) found that ethanol as a solvent led to the maximum curcumin extraction yield as 72% in 1 h at 35°C, compared to the methanol, acetone, and ethyl acetate. Therefore, the extraction process should be optimized based on different extraction parameters such as time, temperature, pressure, the form of solvent, and the ratio of solvent to feed. Gökdemir et al. (139) optimized the ionic liquid bath extraction method by taking independent variables such as time (10–60 min), temperature (25–55°C), and volume of solvent (10–30 mL) and concluded that optimized conditions (60 min, 55°C, and 30 mL) with solvent as the most influential parameter had a higher extraction yield of 2.94%. The extraction of curcumin was optimized by Pan et al. (146) utilizing 80 percent ethanol, 70°C extraction temperature, a liquid-to-material ratio of 20, and a 3 h extraction time to get the highest yield (56.8 mg/g) of curcumin.

**Table 4:** Some recent studies on extraction, isolation, and quantifications of curcumin.

**Sample types**	**Extraction methods**	**Extraction conditions**			**Extraction yield (%)**	**Identification and quantification methods**	**Column type**	**Conditions for identification and quantification**			**Concentration of compound (mg/g dry weight)**	**References**
		**Solvent type**	**Time (min)**	**Temperature (^°^C)**				**Column/plate dimension (mm × mm)**	**Particle size (μm)**	**Injected volume (μL)**		
Powder	Surfactant-free microemulsion (SFME) extraction	Triacetin: Ethanol: water (36:24:40)	-	-	0.921%	HPLC	C18	150 × 2.1	0.03	10	9.21 ± 0.32	(140)
Powder	Refluxing	Dichloromethane	60	-	81.81–86.36	Thin Layer Chromatography (TLC)	-	100 **×** 200	-	-	818.1–863.6	(138)
Powder	Ionic liquid bath	1-butyl-3-methylimidazolium bis(trifluoromethylsulfonyl) imide	10–60	25–55	0.76–2.94	HPLC (Agilent 1100)	C18	150 **×** 4.6	5	20	29.4	(139)
Powder	Dissolving	Ethanol (95%)	10,080	Room temperature	15	Column Chromatography	-	-	-	-	150	(144)
Powder	Soxhlet method	Ethanol	480	70	12.67	Column Chromatography	-	-	-	-	788	(129)
Powder	Soxhlet method	Petroleum ether	60	-	1.55–5.163%	UHPLC	C 18	100 × 2.1	1.7	10	6.58 ± 0.023–21.31 ± 0.301	(66)
Powder	Enzyme-assisted ionic liquid extraction	*N,N*-dipropyl ammonium and *N, N*-dipropylcarbamate	120	Room temperature	1.48–3.95	HPLC (Smartline, Knauer, Germany)	C 18	250 × 4.6	0.45	-	57.3	(128)
Powder	Subcritical water extraction	Water	60	140	76	HPLC	C 18	250 × 4.6	0.45	-	38	(137)
Powder	Subcritical solvent extraction	Water	120	60	10.49–13.96	HPLC Agilent system (1200 series, Agilent Technologies, Santa Clara, CA, USA)	C 18	150 × 4.6	5	20	49.4	(143)

The most commonly used extraction process is the traditional method (Figure 8B) due to its low cost of operation and simple handling, although it has many disadvantages, such as high-temperature operation, longer extraction time, and extensive solvent usage. Modern extraction techniques (Figure 8B) such as ultrasound-assisted extraction (UAE), microwave-assisted extraction (MAE), enzyme-assisted extraction (EAE), and subcritical extraction methods can be used to improve the efficiency and yield of the extraction process (67, 127, 141–143, 147, 148). Most curcumin extraction adopted the traditional method with few exceptions, as shown in Table 4. Kwon and Chung (143) showed that subcritical solvent extraction provided a maximum yield of 13.58% at 135°C/5 min with water/ethanol mixture (50:50, v/v) as a solvent whereas the Zhou et al. (149) reported the optimization of microwave-assisted extraction of curcuminoids from *Curcuma longa* using 69% ethanol, 21:1 liquid:solid ratio and microwave time of 55 s with a yield of 28.97 mg/g rhizomes powder. Among different modern extraction processes, enzyme-assisted ionic liquid extraction and surfactant-free microemulsion (SFME) extraction achieved a yield of 5.73 and 0.76–2.94%, respectively (128, 140). Water used as a solvent in the subcritical extraction process has reduced the toxicity of the organic solvent and serves as a strong substitute for the organic solvent since it is safe and easily available (137, 143). This method also increases the safety of the extract as no organic solvent has been used and can be applied to the food system for use. Subcritical water extraction can also be used as a safer alternative to traditional extraction techniques.

The next step after extraction is the isolation and characterization of bioactive compounds (Figure 8C). Usually, this process is followed by many chromatographic techniques such as column chromatography, TLC, and HPLC coupled with diode array detection, etc. These techniques have been used separately or in combination to characterize unique bioactive compounds (141). Very few scientists have used column chromatography techniques to identify and quantify curcumin. The HPLC system developed by Agilent Technologies is being used by researchers for identification purposes. It is commonly used for the isolation and quantification of bioactive compounds contained in the biological extract (136, 141). Mass spectrometry (MS/MS) techniques can be used to characterize a wide variety of bioactive compounds. However, several analytical methods can be used to improve purity. These novel technologies are ultra-performance liquid chromatography and NMR spectroscopy which can be used to improve the purification and characterization process. Furthermore, mass spectrometry with electrospray ionization can be used to gain further insight into the structure and nature of bioactive compounds to characterize them.

The knowledge mentioned above will potentially benefit researchers, scientists, and industrial people employed in the field of food science. The information addressed will provide them with detailed ideas on the preparation of the sample, the selection of the extraction method, or the selection of suitable solvents to achieve the highest yield. In addition, this section would also be useful for researchers of the above area of interest in selecting the required purification technology as well as the proper identification of the desired bioactive compounds with better quantification. The method that demonstrated the maximum yield to be recommended for the extraction of curcumin from the turmeric and should be used to fortify the food product with improved immunological activity. For the people who are at risk of coronavirus when no drugs are available worldwide, these developed foods for well-recognized immunological functions in humans that have already been clinically demonstrated can be highly recommended for this issue.

## Developed Functional Food Associated With Curcumin and Its Relation With the Immune System and COVID-19

Today, modern consumers have become more conscious and aware of their well-being. As a result, consumers pay more attention to those foods which contain specific ingredients that are capable of affecting their health as well as the different physiological functions. Thus, with this approach in mind, functional foods are successfully formulated using nutraceuticals and bioactive compounds to fulfill the nutritional as well as physiological needs of consumers (25, 150). During the past 20 years, studies have reported many bioactive compounds as ingredients for the production of functional food, such as polyphenols, phytosterols, vitamins, and minerals (22–25, 151, 152). Curcumin is also regarded as an important bioactive compound among these bioactive compounds, which has been identified and extracted from *C. longa* plants (128). The potential health benefits of this polyphenolic compound have also been clinically demonstrated. Based on suitability, curcumin was used for the development of functional food in its free form or encapsulated form, as shown in Table 5. Curcumin is used as a natural ingredient that offers a distinctive color and flavor profile, in addition to potential health benefits during the development of functional foods. Such functional foods can help to boost immunity in humans due to the immunomodulatory properties of curcumin, which has already been addressed in previous sections and thus can help people fight against the COVID-19 virus if such foods are recommended for oral consumption. The following sections addressed some of the important functional foods that have been developed using curcumin as functional ingredients.

**Table 5:** Some recent works on the development of different functional foods using curcumin.

**Source**	**Employed method**	**Amount of curcumin used**	**Developed food**	**Remark**	**References**
**(A) Cereal-based functional foods**					
Pure curcumin powder	Direct	114 mg/bread	Bread	Curcumin added bread significantly reduced the low-density cholesterol and risk of cardiovascular diseases.	(153)
Turmeric powder	Direct	100 mg/g custard powder	Corn starch custard powder	The microbial contamination of custard was reduced more than the control sample.	(154)
Turmeric powder	Direct	50–75 mg/g wheat flour	Crackers	By adding turmeric powders the taste of the crackers improved because they had normally a pleasant turmeric taste. Overall findings indicate that turmeric powders were functional food additives with high phenolic content, antioxidant capacity, and bio-accessibility.	(155)
Turmeric powder	Direct	10 mg/g maize flour	Kokoro (Nigerian snacks)	The sensory properties of Kokoro in terms of color, aroma, taste, texture, and overall acceptability were increased.	(156)
Pure curcumin powder	Encapsulation (Liposome)	730 mg/g lyophilized liposome	Cake	The liposomes were lyophilized by pro-liposome hydration and the curcumin encapsulated remained stable after 70 days of storage. The results indicate that hardening and chewing were reduced and the color of the agglomerated cornstarch cakes was intensive and uniform.	(157)
Turmeric powder	Direct	5 mg/g flour mixture	Biscuit	Turmeric powder had the highest positive effect on the antioxidant properties of the biscuits.	(158)
Turmeric powder	Direct	50 g/kg	Whole grain wheat flour (WGF) pasta	A formulation, TP3 observed the highest total phenolics content (534.46 ± 1.93 mg kg^−1^), DPPH∙ (5.70 ± 0.10 g kg^−1^ of Trolox Equivalent (TE), and ABTS (9.02 ± 0.58 g kg^−1^ of TE), with high retention of antioxidant capacity after cooking; it is suggestive that wheat fiber and bioactive compounds from turmeric may be conjugated as a natural ingredient in pasta.	(159)
Turmeric extract	Encapsulation	10–80 mg encapsulated curcumin/g flour mixture	Extrudate product	Extruded cereals containing encapsulated turmeric extract also demonstrated strong antioxidant activity when examined using ABTS and DPPH scavenging methods.	(160)
Pure curcumin powder	Encapsulation	-	Bread	The findings indicate that, when its concentration was >0.035%, curcumin microcapsules had preservative effects on food even though it was boiled. In comparison with free curcumin, not only were microcapsules better soluble and hot, but mold spores decreased by 34.5 × 2.5% to 52.3 × 4.1%.	(80)
**(B) Dairy-based functional foods**					
Turmeric powder	Direct	1–2 mg/g butter	Cow milk Butter	Turmeric extract powder had a major effect on mold, coliform, and total microbial counts in all doses (*p* ≤ 0.05) and reached 17, 18, and 960 CFU/mL at the maximum dosage level, respectively.	(161)
Turmeric powder	Direct	0.1–0.5 mg/mg dairy product	Cow milk-based dairy product	Turmeric extract powder had a major effect on mold, coliform, and total microbial counts in all doses (*p* ≤ 0.05) and reached 17, 18, and 960 CFU/mL at the maximum dosage level, respectively.	(162)
Turmeric powder	Direct	0.1–0.3%	Soft cheese	Results indicated that as the concentration of the turmeric powder increased, the total bacterial count as well as coliform count decreased compared with control treatment which showed the highest total count after 9 days of storage at 5 ± 2°C.	(163)
Pure curcumin powder	Nano-emulsion	25 mg/g coating mixture	Skim milk	When it was applied to milk, the lipid oxidation could be prevented by either nanostructure, as demonstrated by an insignificant change in the color of fortified milk after 5 days.	(82)
Turmeric extract	Nano-emulsion	1–10 mg/mL milk	Milk	Turmeric nano-emulsion in milk was able to preserve curcuminoids during gastric digestion and to effectively release them during intestinal digestion as compared to unencapsulated turmeric extract, possibly due to the low solubility of the latter and the degradation of curcumin in the neutral-alkaline medium.	(83)
Pure curcumin powder	Direct	230.8 ± 6.5–232 ± 1 μg/mL milk	Milk	The bioaccessibility of curcumin evaluated using the *in vitro* gastrointestinal (GI) tract was ~40%, which was due to some chemical degradation and binding of curcumin which decreased its stability and solubilization.	(73)
Pure curcumin powder	Nano-emulsion	10–20 mg/g ice cream	Ice cream	The incorporation of turmeric nano-emulsion was a feasible option for reducing the use of artificial dyes, as ice creams demonstrated similar physicochemical and rheological properties.	(164)
Pure curcumin powder	Nano-emulsion	3 mg/g nano-emulsion	Milk	Cut-NEs-fortified milk was demonstrated substantially lower lipid oxidation than control (unfortified) milk and milk containing curcumin-free nano-emulsions.	(81)
Pure curcumin powder	Nano-emulsion	2.4 mg/g ice cream	Ice cream	Release kinetics results indicated that in simulated gastrointestinal digestion, nano-emulsion was stable against pepsin digestion (5.25% release of curcumin), while pancreatic activity resulted in 16.12% release of curcumin from nano-emulsion. No major difference was found in the scores of the sensory attributes between the control and the ice cream prepared with the nano-emulsion of curcumin.	(165)
**(C) Miscellaneous developed functional foods**					
Pure curcumin powder	Direct	100–300 mg/kg meat	Lamb meat	Total fat in meat was substantially lower in the T200 and T300 groups than in the control group. Total SFAs were slightly lower in the T300 group than in the control group, while total PUFAs were higher. No significant differences were observed between groups with respect to total monounsaturated fatty acids (MUFAs).	(166)
Pure curcumin powder	Gel Form	3.4 mg/g gel	Meat pâté	When pork backfat was partially or fully replaced by curcumin-loaded gel in pâtés, a noticeable protective effect of curcumin against lipid oxidation was observed during refrigerated storage.	(167)
Turmeric powder	Direct	20–60 mg/g *Hibiscus sabdariffa* powder	Zobo (Traditional *H. sabdariffa* beverage)	The addition of turmeric to zobo had increased the nutritional quality of zobo.	(168)
Turmeric powder	Direct	5–15 mg/g coating flour	Chicken nugget	Curcuma flour fortification did not affect the water-holding ability, tenderness, protein, and fat content of chicken nuggets (*P* > 0.05), but increased the vitamin E and curcumin content of chicken nuggets (*P* < 0.05). Sensory test results showed that the fortification of curcuma flour did not affect the acceptability of the sensory characteristics of the chicken nugget.	(169)
Turmeric powder	Direct	7–22 g/smoothie	Fruit smoothie	The development of a functional beverage with 14 grams of turmeric was deemed significantly more appropriate with the provision of health information and resulted in a substantial increase in antioxidant ability and polyphenol content.	(170)
Turmeric extract	Nano-emulsion	10 mg/mL prepared emulsion	Canned Ham	The canned ham incorporating the turmeric nano-emulsion powder obtained the same overall acceptability score as the control and demonstrated only mild yellowing.	(171)

### Cereal-Based Product

Cereal-based products, such as bread, pasta, cookies, and cakes, are used by most people around the world as their main source of energy and nutrients. While fungal growth is a major problem in cereal-based food products, the interest in bakery products continues to rise day by day due to their nutritional properties (157, 158, 172, 173). In addition, natural polyphenols such as rice or wheat bran, grape seed extract, fruit pomace powder, ginger, and turmeric are used in bakery products to improve the antioxidant function of the food. The addition of these ingredients to the dough greatly improves the content and antioxidant potential of breads and biscuits (153, 155, 174).

Since curcumin has a variety of other activities that promote health, it is mainly used in bakery products (Table 5A). In one of the earlier approaches, the highest percentage of turmeric powder (8%) observed the highest curcumin (203 mg/kg) and RSA-DPPH activity (45%) in the cake, but it showed the worst results in terms of the rheological properties (83). The authors concluded that 6% addition of turmeric gave the best results in regard to the rheological and sensory properties of the cake. Turmeric flowers are reported to be a rich source of essential oils like *p*-cymen8-ol, hence in this context Azmi et al. (175) employed aqueous extracts of fresh turmeric flowers (5–20%) in cookies to enhance its functional value. Lower antioxidant activity (DPPH assay) was observed in cookies prepared with higher extraction levels of turmeric flowers (20%) and it was attributed to the baking process that led to the degradation of heat-sensitive antioxidant compounds. The authors recommended that 5% and 10% incorporation levels of flower extracts can be the best formulations for bakery products. Ferguson et al. (153) developed two phytosterols-enriched breads in an experiment: (1) with curcumin, and (2) without the addition of curcumin. They found a substantial reduction of low-density cholesterol and risk of cardiovascular disease in the daily intake of those two breads with or without the addition of curcumin (114 mg curcumin/bread). After adding curcumin to crackers enhanced the antioxidant activity and phenolic contents of crackers, as well as the bio-accessibility, flavor, and taste (155). Curcumin can also be added in encapsulated form. The encapsulated curcumin reduced the hardness and chewiness as well as the homogeneous yellow color of the cakes developed with the agglomerated cornstarch (157). It was also found that encapsulated curcumin increased the shelf life of the breads by reducing the growth of the mold spores (80). Adegoke et al. (158) added 5 mg of curcumin per gram of wheat flour to make cookies. The results of their research indicated that curcumin had the strongest positive effect on the antioxidant properties of biscuits. These results indicate that the addition of curcumin to the composite flour would enhance the functionality and nutritional composition of the baked food products. Curcumin-rich turmeric (*Curcuma longa*) powder extracts were used as a natural antioxidant in the preparation of biscuits by Hefnawy et al. (176) along with carrot (*Daucus carota*) and grape (*Vitis vinifera*) leaf. Besides this, turmeric from 1 to 5% was used to improve the color, increase the fiber content and increase the antioxidant properties of pasta while retaining the technological attributes (159).

It can be thus concluded that bakery products can be the perfect medium for the delivery of curcumin to the human body. Since curcumin has anti-inflammatory, anti-oxidant, anti-cancer, immune enhancement effects, it can boost the functionality of food without affecting the sensory properties of bakery products. The immunological activity of curcumin is linked to macrophages, B, and T lymphocytes. Curcumin can enhance immunological activity by a variety of mechanisms, such as the regulation of cytokines and several transcript factors (114). These curcumins are added to bakery products, such as bread, biscuits, and cake, which can quickly boost immunity in humans after consumption due to the increased immunity of curcumin.

### Dairy-Based Product

Dairy-related products were used, in particular for functional dairy products that make up 40% of the global demand for functional food. The global demand for dairy products is very competitive and this worldwide dairy demand will hit US$ 13.9 billion by 2021 (177), without considering the traditional dairy products such as buttermilk, kefir, etc. Dairy products are widely appreciated and flexible and are important for any population group regardless of height, weight, and age. The key benefit of the use of dairy products is the prevention and treatment of many diseases such as diabetes, malnutrition, cancer, and hypertension affecting the world's population (178). Functional ingredients most widely used in the development of functional dairy products include vitamins, probiotics or prebiotics, bioactive compounds, minerals, etc. (177). In bioactive compounds, polyphenols are known as functional food formulations due to their various health benefits such as antioxidant, antimicrobial, antidiabetic, anti-allergenic, anti-inflammatory operation, etc. (71, 152). Curcumin, a natural polyphenol of turmeric rhizome with anti-inflammatory, anti-oxidant, anti-cancer, and immunosuppressive effects, is used mainly in the development of dairy products. Further, it is also observed that milk and milk products are considered as the best suitable media for curcumin as the presence of fat (triglycerides) enhances the solubility of curcumin; and hence its bioavailability in the body.

Curcumin extracted from turmeric has been used in cow's milk butter to increase its functionality (161). Asadaii et al. found that the addition of curcumin in the range of 1–2 mg/g butter improved nutritional and antimicrobial activity by removing mold, coliform, and total microbial counts (161). Furthermore, the addition of curcumin to cow's milk also increased the calcium content and antioxidant activity throughout the storage period (162). Nano-emulsion is a novel technique designed to increase the bioavailability of curcumin. Adding 25 mg curcumin/g encapsulated material or 1–10 mg curcumin nano-emulsion/mL milk to milk minimized lipid oxidation and also effectively released during intestinal digestion (82, 83). In a recent investigation, Hasneen et al. (179) demonstrated that the total phenolic content of yogurt was improved by the incorporation of 1% turmeric extract. Similarly, the radical scavenging activity (RSA) % of both skim milk yogurt and cast Kariesh cheese were significantly enhanced by turmeric. Similarly, in another approach, Ricotta cheese supplemented with curcumin was observed to enhance organoleptic properties, phenolic compounds, and antioxidant activity (180). Additionally, several studies have also shown that the addition of turmeric powder led to inhibit the growth of coliforms and other bacteria in milk-based products especially, cheese during the storage period (163, 180, 181).

Curcumin has also been applied to dairy products, such as ice cream, by several researchers. In an experiment, Borrin et al. (164) added 10–20 mg of pure curcumin per g of ice cream to investigate the effect of curcumin and found that curcumin could be a better alternative to artificial dye, and prepared functional ice cream also showed similar physiological properties compare to control. The curcumin emulsion incorporated into the ice cream was stable against pepsin digestion and there was no major difference between the sensory properties of curcumin added and the control of ice cream (165). Further, the addition of curcumin (160–350 ppm) not only improved the sensory attribute of ghee but also contributed to its antioxidant potential, as found by Lodh et al. (182). On the other hand, the turmeric powder containing curcumin was used to prepare soft cheese which improved its oxidative stability and microbiological quality (163). A detailed summary of the development of some dairy functional foods using curcumin has been shown in Table 5B.

Based on the above findings, it can be concluded that the addition of curcumin could improve the consistency, nutritional value, sensory characteristics, and shelf life of functional dairy products without the addition of any artificial dye or other substances. Due to its antimicrobial and immunity-boosting properties, curcumin can be used in a variety of ways in dairy products. Dairy products are a rich source of vitamin-B complex, protein and calcium and the addition of curcumin to increase the nutritional value of dairy products and these dairy products, due to their enhanced nutritional properties and the availability of vitamins and minerals, can be eaten more and more to create a powerful protection mechanism within the body to defend against the novel coronavirus.

### Other Functional Food Product

The health benefit of functional foods is derived from the bioactive compounds, such as phytochemicals, vitamins, and peptides, found naturally in them, formed during processing, or extracted from other sources and added to them (62, 151). The functional food other than cereal-based or dairy-based that are highly demanding are meat-based and fruit and vegetable-based products (Table 5C). Though meat is highly nutritious but presence of certain compounds affects negatively on the human health. Therefore, the functional food concept gives an excellent opportunity to improve the functionality of meat products (24). In functional meat production, generally synthetic and natural GRAS (generally recommended as safe) ingredients were used to improve the end products' safety and quality. Therefore, plant bioactive compounds because of their health benefits are now are of great interest. The incorporation of these bioactive compounds improves the nutrient and functionality of meat products (22). The increasing awareness regarding the nutritional compounds and health benefits of fruit juices increased consumer demand toward healthy fruit or vegetable-based beverages increased as compared to carbonated drinks. Fruit juices, which are naturally rich in bioactive compounds with health-promoting and disease-reducing properties, are important contributors to human nutrition (72). Fortification of fruit juices with natural bioactive compounds such as polyphenols (183), active peptides (184), vitamins (185) etc., has been investigated for several years.

However, there is very little literature available on the incorporation of curcumin to develop functional meat and fruit-based food products. The curcumin was added to lamb meat with an aim to reduce the fat in meat and it was observed that the addition of 100–300 mg curcumin/g meat reduced the saturated fatty acid without affecting PUFA and MUFA (166). A similar effect was observed in meat pâté where pork fat was totally or partially replaced by the curcumin hydrogel and lipid oxidation was also reduced (167). During modified atmospheric packaging of fresh lamb sausages, turmeric extract rich in curcumin slowed the lipid oxidation and generation of related volatile compounds as well as improved the antioxidant capacity (186). Moreover, except for the yellow color, none of the physico-chemical parameters of the product were greatly influenced by the addition of turmeric extract. The authors concluded that the addition of turmeric extract can replace sodium erythorbate up to 500 ppm. In another experiment, Sujarwanta et al. (169) found that direct incorporation of 5–15 mg curcumin/g flour improved the vitamin-E and curcumin content without affecting the sensory characteristics of the chicken nugget. Direct turmeric was also added 7-22 g per g of smoothie to study the effect of turmeric on smoothie by 138. In this experiment, the author found that the addition of 14 grams of turmeric to the beverage improved the functionality of the smoothie with a significant increase in polyphenol content and antioxidant capacity. A substitution of wheat flour with turmeric powder (up to 4%) observed good antioxidant activity as well as acceptable sensory scores with normal wheat bread; thus, authors concluded that breads incorporated with turmeric powder can be developed as a health-promoting functional food (187). Zenzer et al. (188) performed a randomized, single-blind study and concluded that compared to control, spice-based beverages containing turmeric increased p-PYY (plasma-peptide tyrosin-tyrosin) and lowered the “desire to eat” and “prospective consumption (quantity of food wanted to it)” in a healthy human.

Curcumin has many health promoting activities and is an outstanding compound. Increasing customer demand for food and beverages with health-promoting nutrients and immunity boosting components has been met by developing functional food and drinks. This improves the health of humans by adding turmeric to regulate the fat content of some items. Curcumin not only improves the color of meat products but also increases the content of vitamin-E in poultry nuggets, which allows the human body to receive more vitamin-E. The increased availability of functional foods and drinks has a long-term impact on human health. These rich curcumin foods can enhance immunity in the human body, reducing the possibility of an invasion of coronavirus.

## Limitations and Advances in the Use of Curcumin in Rational Drug Therapy

Curcumin is extensively employed in ayurvedic herbal remedies from ancient times. Several *in-vitro* and *in-vivo* studies have shown that curcumin possesses the chemo-preventive and chemotherapeutic agents for colon, skin, oral and intestinal cancers (87, 117). However, in humans phase I/II clinical trials have revealed that curcumin exhibited the low bioavailability. Owing to poor bioavailability and rapid metabolism, the clinical efficacy of oral curcumin may be lower than *in-vitro* studies. It is reported that *in-vitro* the therapeutic potential of curcumin has been reported at micromolar range concentrations, whereas after oral intake the plasma concentration is in the nanomolar range (189). Recent technological developments including adjuvants, liposomes, phospholipid complexes, micelles, and nanoparticles are being evaluated to increase the oral bioavailability of curcumin. It is suggested that for the instability and weak pharmacokinetic profiles of curcumin, the β-diketone moiety is responsible (13). Hence, researchers have performed structural modification in curcumin to prepare the analogs without the β-diketone moiety. Predominantly, mono-carbonyl analogs of curcumin (MACs) have been reported to have enhanced stability *in-vitro* and an improved pharmacokinetic profile *in-vivo* and some of them have been intensively studied in order to develop novel drugs (13). The majority of studies often do not mention the exact amount of curcumin employed, and it is variable in commercial preparations. Additionally, it exists in different forms that also differ in biological potencies (190). *In-vitro* results suggested that curcuminoids inhibit collagenase, hyaluronidase, and elastase (90, 191). Curcuminoids have potential in cosmeceuticals as antioxidant and skin lightening agents.

Sundar et al. (192) developed 3-aminopropyltriethoxy silane (APTES) coated magnetite nanopowders as carriers for curcumin as an anticancer drug. It was prepared through the modified controlled chemical co-precipitation method employing oleic acid as the apt surfactant to achieve well-dispersed ultrafine spherical Fe_3_O_4_ magnetic nanoparticles. Within 1 h about 15% drug was released while 80% drug release was observed in 48 h, showing its promising application for *in-vivo* trials. Similarly, Mai et al. (193) fabricated polylactic acid (PLA) microcapsules as novel drug delivery systems using curcumin as a model drug by using an electrospray technique. The drug-loaded microcapsules had significant biocompatibility, low cytotoxicity, and had shown excellent anti-bacterial activities. The authors concluded that it has a potential for a wide range of applications in medical fields including drug delivery (193). To sum up, it can be stated that along with previously used technologies like liposomes, microencapsulation as well as nanoencapsulation are the newly emerging viable alternatives that have been shown to deliver therapeutic concentrations of potent chemo-preventive including curcumin and other polyphenols into the systemic circulation.

## Future Prospective and Research Opportunities

The major purpose of enrichment or fortification of the food product is to achieve the desired properties such as increase nutrient composition, influence physicochemical characteristics, reduce oxidation, improve sensory characteristics, and enhance product shelf life. Based on previous research on the biological activities of curcumin, it has been found that there is a great potential for it to be used as a functional ingredient in the development of functional foods. After a thorough review, it may be suggested that encapsulated curcumin is more effective than direct incorporation. Directly added curcumin can be degraded by the digestive enzymes whereas in presence of suitable coating material encapsulated curcumin can withstand the effect of digestive enzymes and their direct release in the gastrointestinal tract can increase their bio-availability. Therefore, food scientists and researchers are recommended to work more on the encapsulation of curcumin and study the different properties of the encapsulated curcumin. Besides this, as curcumin has a positive impact on the human immunity system, it is required to do more and more studies on it. Because one of the main reasons for coronavirus spread was poor human immunity. Hence, it is required to study the effect of curcumin on humans as till now it has been done *in-vitro* or in an animal model. Further, the best way to consume curcumin is by incorporating it into food. From this review, it was found that many researchers have incorporated curcumin into the food matrix to develop functional food. However, none of them has done the immunological effect of curcumin added food products on human or animal models or *in-vitro*. Consequently, it is a great opportunity for researchers or food scientists to perform those experiments to collect more strong evidence of the effect of curcumin against COVID-19. In addition, there is a need for commercialization and awareness regarding the health beneficial activities especially the immunological effect of curcumin added food is needed. Therefore, it is humble advice from the authors to the respective country government and the concerned authorities of the World Health Organization (WHO) to spread the health benefits of curcumin and its advantages against novel coronavirus, in order to control the spread of the virus.

## Concluding Remarks

The purpose of the present review is to discuss about the different extraction, isolation, and quantification methods of curcumin and its potential application in respiratory diseases such as COVID-19. Several methods were found for extraction, isolation, and quantification. But the subcritical water extraction method and HPLC is the most effective method with a higher extraction yield. In the development of functional food, curcumin was successfully added to different food matrices to develop functional food. These functional foods being rich in curcumin can enhance the immunity to fight against novel coronavirus. However, further progress is still essential, especially in terms of the development of functional foods. Additional information is required on the effects of food processing and storage conditions on the biological potential of curcumin. Further, healthcare organizations across the globe need to spread awareness regarding the immunomodulatory properties of curcumin and people should consume these functional foods in order to improve their immunity to fight against various pathogens including the SARS-CoV-2. Together, by consuming healthy nutritious food and following the WHO guidelines, we can win the fight against coronavirus.

## Author Contributions

DV has conceptualized, interpreted, corrected, and technically sound final versions of the manuscript. ST has compiled literature for manuscripts. MT, SS, AP, HV, and AG have interpreted the manuscript, corrected it, and made it scientifically sound for the final version. MC-G and GU have read final versions of the manuscript. NC has provided suggestions, medical expertise, and corrections for the final version. CA has provided technical suggestions, corrections, and permissions for the finalization of the manuscript. PS has read and approved permissions for the finalization and submission of the manuscript. All the authors approved the submission of this manuscript.

## Conflict of Interest

The authors declare that the research was conducted in the absence of any commercial or financial relationships that could be construed as a potential conflict of interest.

## Publisher's Note

All claims expressed in this article are solely those of the authors and do not necessarily represent those of their affiliated organizations, or those of the publisher, the editors and the reviewers. Any product that may be evaluated in this article, or claim that may be made by its manufacturer, is not guaranteed or endorsed by the publisher.
